# Assessment of green development level performance in G20 countries: A dynamic evaluation framework

**DOI:** 10.1016/j.heliyon.2024.e37622

**Published:** 2024-09-07

**Authors:** Tarifa Almulhim

**Affiliations:** Department of Quantitative Methods, School of Business, King Faisal University, Al-Ahsa, 31982, Saudi Arabia

**Keywords:** Green development level, G20, Dynamic technique for order preference by similarity to ideal solution, Principal component analysis, Dynamic entropy weighting

## Abstract

To achieve sustainable development goals and foster future prosperity globally, the promotion of green development is essential. Nevertheless, persistent regional disparities in green development among the G20 nations stem from differing economic and environmental conditions in dynamic contexts. In light of these conditions, it is essential for governments to evaluate their country's current green development performance in comparison with higher-performing nations and to learn from their experiences. This study introduces a new dynamic evaluation framework that integrates the importance of evaluation indicators using the dynamic entropy weighting method, a Dynamic Technique for Order Preference by Similarity to Ideal Solution method, and principal component analysis. This framework serves as a tool for G20 countries to assess their green development levels between 2015 and 2021. A key contribution of this study is the facilitation of the G20's ability to implement relevant reform measures and monitor green development progress in a dynamic environment. This framework can serve as a model for similar evaluations in other regions globally. The study's main findings indicate that G20 countries with high levels of green development possess strong economic foundations and prioritise the balanced development of their economies, societies, environments, and innovation.

## 1 Introduction

Achieving a substantial level of green development is deemed paramount to all nations worldwide in the pursuit of sustainable development, thereby contributing to enhanced future prosperity. This endeavour necessitates prioritising the quality and efficiency of development, underpinned by the judicious utilisation of resources and comprehensive environmental preservation within economically feasible frameworks. A systematic and reasonable evaluation of green development performance levels is important for governments in formulating sustainable development goals and plans. Specifically, within the context of the G20, evaluating the green development performance of member states is crucial for achieving the United Nations Sustainable Development Goals by 2030. The G20, a prominent international economic consortium, represents 80 % of the global gross domestic product (GDP) and encompasses 60 % of the world's population [1]. Furthermore, directing attention towards assessing the level of green development performance among G20 countries is significant, given that it comprises the most industrialised nations, which are conventionally presumed to exert the greatest influence on environmental degradation [2].

To date, little progress has been made in contemporary academic research focusing on the evaluation of green development levels. Li et al. [3] formulated a framework for green development indicators spanning various dimensions, including the living environment, economic growth, pollutant treatment and utilisation, innovation potential, and ecological efficiency. Utilising the S-type cloud model, they assessed the green development status within the Beijing–Tianjin–Hebei region of China. Wang et al. [4] contributed significantly to the analysis of green development by incorporating health-related metrics and constructing indicators that address the economic, environmental, and health aspects. Subsequently, they applied the weight cloud model to analyse the green development levels observed among China's listed mineral resource companies. Han et al. [5] delineated a comprehensive set of indicators based on the economic, resource, and health dimensions. Employing a multi-criteria decision-making (MCDM) approach, specifically, the entropy weight method, along with spatiotemporal analysis techniques, they assessed the green development status of the Association of Southeast Asian Nations member states. Similarly, Babacan et al. [6] utilised an MCDM method known as stepwise weight assessment ratio analysis (SWARA) to evaluate green economy performance across Organization for Economic Cooperation and Development (OECD) countries.

Previous studies have developed evaluation systems encompassing various dimensions, including economics, environment, resources, and health. However, these frameworks have overlooked the social implications inherent in assessing the level of green development at the national level, such as the human development index (HDI), and innovation-related aspects, such as the innovation ability index and research and development (R&D) expenditure. Drawing on the pertinent literature and considering the indicators influencing green development performance, we propose an urgent need to build a comprehensive evaluation framework structured around dimensions encompassing environmental, economic, social, and innovation aspects.

In the context of assessing green development levels, the evaluation process is recognised as an MCDM challenge given the typically extensive, intricate, and conflicting nature of evaluation indicators [5,6]. Recently, predominant evaluation methodologies such as the Technique for Order Preference by Similarity to Ideal Solution (TOPSIS) [7,8], the entropy weighting method (EWM) [5], and SWARA [6] have been widely adopted in practice. However, these methods overlook the temporal dynamics of evaluation indicators, thereby impeding a comprehensive capture of dynamic shifts within the subjects under evaluation [8]. Achieving a thorough vertical assessment that accounts for temporal variations necessitates a dynamic evaluation framework. Such a framework must reflect how the performance of the evaluation system evolves over different points in consecutive periods, thereby affecting the final evaluation outcome. In other words, it is essential to incorporate a time variable into the evaluation process to capture these temporal dynamics accurately. Furthermore, a universally accepted standard for the evaluation and benchmarking process of national development levels remains elusive, particularly concerning specific steps, weighting, ranking and clustering [9], specifically within dynamic contexts. Hence, there is ample opportunity to devise a more appropriate adaptable dynamic framework for countries to assess their green development levels performance through a three-step process involving weighting, ranking, and subsequent clustering.

Based on the research gap in the assessment of green development performance at the national level, this study comprises the following stages: First, according to the relevant requirements and objectives for green development at the national level and given the deficiencies of existing evaluation indicators, this study constructs an assessment indicator system that satisfies the requirements of green development performance by utilising environmental, economic, social, and innovation dimensions. Second, this study proposes a new dynamic evaluation framework that integrates dynamic EWM (DEWM), dynamic TOPSIS (DTOPSIS), and principal component analysis (PCA), which we call DEWM–DTOPSIS–PCA. The proposed dynamic evaluation framework offers effective methods for measuring the importance of multiple indicators, ranking various countries, and clustering the countries being studied. Third, the proposed dynamic evaluation framework, which is based on panel data, is used to dynamically evaluate the national green development level of G20 countries for the period between 2015 and 2021. Finally, we propose management suggestions and offer practical implications.

The remainder of the paper is organised as follows. Section 2 presents a theoretical literature review of the evaluation methods. Section 3 introduces the research methods, data sources, and materials used. Section 4 presents the analysis and summarises the empirical results. Section 5 discusses the results and implications of this study. Finally, the conclusions and limitations of this study are presented in Section 6.

## 2 Literature review

Despite the availability of MCDM and other statistical methods, their application in evaluating green development levels is limited (see Table 1). Previous research has developed evaluation systems encompassing various dimensions such as economic, environmental, social, and innovation (Table 1). However, these frameworks typically lack comprehensive evaluations that integrate all four dimensions—environmental, economic, social, and innovation. Furthermore, prior studies have often overlooked the social implications inherent in assessing green development at the national level, such as the Human Development Index (HDI), and innovation-related aspects, including the Innovation Ability Index and R&D expenditure. Previous research has predominantly relied on single evaluation methods, often neglecting dynamic contexts (Table 1). The following paragraphs provide a comprehensive review of various evaluation processes that incorporate dynamic contexts.

**Table 1:** Related studies.

Study	Application areas	Evaluation dimensions				Methods	Dynamic context
		Environmental	Economic	Social	innovation		
Alfalih & Hadj [2]	Assessment of green technological innovation in G20	Yes	No	No	No	Panel quantile regression	No
Li et al. [3]	Assessment of green development level in China	Yes	Yes	No	Yes	Entropy method	No
Wang et al. [4]	Evaluating green development level of mineral resource-listed companies in China	Yes	Yes	No	No	Cloud method	No
Han et al. [5]	Evaluation of green development level of ASEAN region	Yes	Yes	No	No	spatio-temporal patterns	Yes
Babacan et al. [6]	Evaluating the green economy performance in OECD countries.	Yes	Yes	Yes	No	SWARA Method	No
This paper	Assessment of Green Development Level Performance in G20 Countries	Yes	Yes	Yes	Yes	DEWM–DTOPSIS–PCA	Yes

The weighting process is a significant factor that affects the evaluation results [16]. In recent years, several weighting methods have been used to scientifically determine the weight of each indicator [8,9]. Common methods include the EWM [10,11], standard deviation [12], coefficient of variation [13], and criteria importance through inter-criteria correlation (CRITIC) methods [14]. The EWM is commonly and widely used for weighting indicators [[15], [16], [17]]. It objectively determines the indicator weights according to the information provided by each indicator [10], and the calculation process is straightforward and transparent [16]. In addition, the EWM is based on a measure of uncertainty in information data [15,17]. However, previous research on green development evaluation has predominantly utilised single and comprehensive EWMs to determine indicator weights, as shown in Table 1. Therefore, in this study, the DEWM [5,8,18] is applied to derive the weights of green development performance indicators over time.

Assessing and ranking the level of green performance development is crucial for evaluating and analysing the developmental status across countries. In this context, a method for aggregating multiple indicators and ranking various countries to measure green development-level performance is required. In the literature, a few ranking methods have been popularly used in assessing green sustainability performance, such as TOPSIS [7,8], the VIsekrzterijumska Optimizacija i Kompromisno Resenje [7] and the multi-objective optimisation method [19]. Nevertheless, most previous studies did not consider the overall ranking of a country based on multiple indicators across different years. For example, the ranking is usually computed within a single year, which may negatively affect the comparability of the evaluation results across different years. Therefore, this study adopted the DTOPSIS method [8,18,21] in the ranking process, which simultaneously considers the distances to both positive and negative ideal solutions in dynamic situations [18]. It not only considers the differences in countries' green development level performance but also considers the differences in indicators’ weights over time, which can better reflect the overall changes in assessment.

Zhang et al. [9] highlight that, although any indicator evaluation system may accurately reflect the ranking performance of national development levels, high-ranking countries within this system may not offer actionable guidance for countries performing below par. They emphasise the significance of benchmarking, which is intricately linked to a clustering process designed to categorise countries into distinct clusters. This clustering facilitates the identification of appropriate references, specifically the best in-cluster for each country [9,21]. Consequently, a clustering process is imperative to aid countries within each cluster in identifying suitable benchmarks corresponding to the varying stages of green development performance. This approach not only empowers policymakers to devise contextually relevant strategies but also enables the identification of shared challenges and opportunities among countries in similar clusters [9,21]. Principle component analysis is a popular and effective method for clustering and belongs to a group of factor analysis techniques [21,23]. It is a widely used exploratory data analysis technique employed to represent the information within large datasets into a concise set of ‘summary indicators’ for easier visualisation and analysis [9,23]. By reducing the dimensionality of the data, PCA generates principal components that retain essential information from the original dataset, although with fewer data points [9,22]. It is commonly utilised owing to its ability to enhance data interpretability, retain the maximum amount of information possible, and enable the straightforward visualisation of multidimensional data [9]. In this study, PCA is applied to the clustering process using the first two principal components to visually plot the data in two dimensions, identifying clusters of closely related data points.

## 3 Research methods and materials

### 3.1 A dynamic evaluation framework

Three components are required to develop a dynamic evaluation framework: weighting, ranking, and clustering. This study used a dynamic evaluation framework involving the DEWM–DTOPSIS–PCA method, in which the DEWM provides the weights of each indicator over time for the DTOPSIS to gain a dynamic evaluation and ranking that reflects each country's green development performance over time; PCA is then used to group the countries. The dynamic evaluation framework includes the following eight steps:

Step 1: Collecting evaluation information. Set $A=\left\{a_{1},a_{2},\ldots .a_{n}\right\}$, and set $C=\left\{c_{1},c_{2},\ldots .c_{m}\right\}$, where *n* is the number of evaluated alternatives (or G20 countries) and m is the evaluation indicator. The original data matrix $X={\left(x_{ij}\left(t_{h}\right)\;\right)}_{n\times m}$ is obtained, such that $x_{ij}\left(t_{h}\right)$ represents the actual performance value of alternatives (or countries) $a_{i}\text{,}$ with respect to indicator $c_{j}$ in the year $t_{h}$, where i=1,2,···,n,j=1,2,···,m and h=1,2,···,k*.* Let $x_{ij}\left(t_{h}\right)\geq 0$, the indicators’ weight vector $W=\left\{w_{1},w_{2},\ldots .w_{m}\right\}$, satisfied by $\sum_{j=1}^{m}w_{j}=1$, $w_{j}\in \left[0,1\right]$.

Step 2: Normalising the collected data. The normalised data matrix $Y={\left(y_{ij}\left(t_{h}\right)\;\right)}_{n\times m}$ is obtained using the Z-score method [[20], [21]].

Step 3: Deriving indicator weights. The DEWM method [18] is employed to determine the weight using the following equations:

(1) $$p_{ij}\left(t_{h}\right)=\frac{y_{ij}\left(t_{h}\right)}{\sum_{h=1}^{k}\sum_{i=1}^{n}y_{ij}\left(t_{h}\right)}$$

(2) $$E_{j}=-\left(\frac{1}{\ln\left(k*n\right)}\right)\sum_{h=1}^{k}\sum_{i=1}^{n}p_{ij}\left(t_{h}\right)\ln\;p_{ij}\left(t_{h}\right)$$

Then, the weight of indicator $c_{j}$ is calculated by the following:

(3) $$w_{j}=\frac{1-E_{j}}{\sum_{j=1}^{m}\left(1-E_{j}\right)}$$

Step 4: Constructing a weighted normalised matrix. The weighted normalised decision matrix is computed as $Z={\left(z_{ij}\left(t_{h}\right)\;\right)}_{n\times m}$, where $z_{ij}\left(t_{h}\right)$ is calculated as below [21]:

(4) $$z_{ij}\left(t_{h}\right)=w_{j}\;y_{ij}\left(t_{h}\right)$$

Step 5: Determining the positive ideal $Z^{+}$ and negative ideal $Z^{-}$ solutions for conducting the DTOPSIS method. In this step, the optimal value, denoted as ${z_{j}}^{+}$, and the negative ideal value, denoted as ${z_{j}}^{-}$, of indicator $c_{j}$ are computed as follows [18,21]:

(5) $${z_{j}}^{+}=\max\{z_{ij}\left(t_{h}\right),i=1,..n\;;h=1,..k\}$$

(6) $${z_{j}}^{-}=\min\{z_{ij}\left(t_{h}\right),i=1,..n\;;h=1,..k\}$$

Step 6: Calculating Euclidean distance. The distance between the evaluation values of alternative $a_{i}$ in the year $t_{h}$ and the ideal solution, denoted as ${d_{i}}^{+}\left(t_{h}\right)$, is calculated as follows [18,21]:

(7) $${d_{i}}^{+}\left(t_{h}\right)=\sqrt{\sum_{j=1}^{m}{\left(z_{ij}\left(t_{h}\right)-{z_{j}}^{+}\right)}^{2}}$$

Similarly, the distance between the evaluation values of alternative $a_{i}$ in year $t_{h}$ and the negative ideal solution, denoted as ${d_{i}}^{-}\left(t_{h}\right)$, is calculated as follows [18,21]:

(8) $${d_{i}}^{-}\left(t_{h}\right)=\sqrt{\sum_{j=1}^{m}{\left(z_{ij}\left(t_{h}\right)-{z_{j}}^{-}\right)}^{2}}$$

Step 7: Computing the relative performance values and ranking the alternatives. The relative performance level of alternative $a_{i}$ in the year $t_{h}$, denoted as ${R_{i}}^{*}\left(t_{h}\right)$, is calculated using the following equation [18,21]:

(9) $${R_{i}}^{*}\left(t_{h}\right)=\frac{{d_{i}}^{-}\left(t_{h}\right)}{{d_{i}}^{+}\left(t_{h}\right)+{d_{i}}^{-}\left(t_{h}\right)}$$

Obviously, ${R_{i}}^{*}\left(t_{h}\right)\in \left[0,1\right]$.

The ranking of alternative $a_{i}$ is then based on ${R_{i}}^{*}\left(t_{h}\right)$ values in descending order.

Step 8. Clustering alternatives. Finally, the clustering process proposed by Zhang et al. [9] is used. PCA is applied, with eigenvalues >1 serving as the criterion for selecting several factors. Scores are then computed for each alternative and factor. As described by Zhang et al. [9], these scores for each country are weighted according to the variance explained by each factor and normalised by the total variance. The resulting factors are further weighted to create a composite index, known as the weighted index. Countries are subsequently grouped into similar categories based on a scatterplot, where the weighted index and values of the first factor are used as the x- and y-axes, respectively.

### 3.2 Construction of the assessment indicator system

To assess the level of green development performance nationally, it is necessary to create a comprehensive assessment indicator system for the level of green development performance in G20 countries. This system includes four dimensions – environmental, economic, social, and innovation – with 18 indicators (Table 2).

**Table 2:** Assessment indicator system for the green development level of the G20 members.

Dimensions	Indicator	Unit	Related Documents
Environmental	Forest cover ($c_{1}$)	%	[29,30]
	Arable land ($c_{2}$)	% of land area	[6,29]
	Share of renewable energy ($c_{3}$)	%	[17,25,30,31]
	Ecological footprint ($c_{4}$)	GHA per capita	[8,25,32]
	Energy intensity level of primary energy ($c_{5}$)	MJ/$2017 PPP GDP	[5,31]
	CO_2_ emissions ($c_{6}$)	kt	[5,17,30,31,33]
Economic	Gross national income ($c_{7}$)	USD/Capita	[29,32]
	Agriculture, forestry and fishing, value added ($c_{8}$)	% of GDP	[6,29]
	Foreign direct investment ($c_{9}$)	% of GDP	[8,16,34]
	Gross fixed capital formation ($c_{10}$)	% of GDP	[8,25]
	GDP per capita ($c_{11}$)	constant 2015 US$	[3,29]
	GDP ($c_{12}$)	constant 2015 US$	[8,25]
Social	Life expectancy ($c_{13}$)	years	[5,29]
	Human development index ($c_{14}$)	value between 0 and 1	[6,35]
	Unemployment rate ($c_{15}$)	%	[5,8]
Innovation	Number of patents granted ($c_{16}$)	pieces	[30,33]
	Research and development expenditure ($c_{17}$)	% of GDP	[8,33]
	Innovation ability/high technology exports ($c_{18}$)	% of manufactured exports	[16,34]

The first dimension is primarily concerned with the environmental sustainability of each G20 member. Environmental sustainability is the basis of national green development levels [24,25]. Moreover, negative environmental effects such as pollution and energy consumption pose significant obstacles to sustainable green development [3]. This dimension includes indicators such as forest cover ($c_{1}$), arable land ($c_{2}$), share of renewable energy ($c_{3}$), ecological footprint ($c_{4}$), energy intensity level of primary energy ($c_{5}$), and CO_2_ emissions ($c_{6}$).

The second dimension is related to the economic development of each G20 member. This is described in terms of the current state of economic development. It is necessary to balance the speed and quality of economic growth to achieve green development performance at the national level [26,27]. The indicators considered for this dimension are gross national income ($c_{7}$); agriculture, forestry, fishing value-added ($c_{8}$); foreign direct investment ($c_{9}$); gross fixed capital formation ($c_{10}$); GDP per capita ($c_{11}$); and GDP ($c_{12}$).

The third dimension is related to social development. Green development aims to improve social desires [8]. The indicators considered for this dimension are life expectancy ($c_{13}$), HDI ($c_{14}$), and the unemployment rate ($c_{15}$).

Technological innovation development is selected as the fourth dimension. The innovation development potential usually measures the maturity of green sustainability and promotes national green development performance level [28]. This dimension includes indicators such as the number of patents granted ($c_{16}$), R&D expenditures ($c_{17}$), and innovation ability/high-technology exports ($c_{18}$).

### 3.3 Data collection

In this study, the G20 member countries^1^ are selected because they are at the forefront of advancing the idea of achieving sustainable levels of green development, as outlined in the introduction. Considering the pivotal role of the 2015 Paris Climate Agreement as an indispensable instrument for catalysing successful global green development [36], this study delineated its investigation between 2015 and 2021. This period is selected to coincide with the aforementioned landmark agreement and is delimited by data availability constraints, yielding a comprehensive sample comprising 2,394 observations for 18 indicators over seven years. The data for the indicators are primarily gathered from public databases and assessment reports, such as the World Bank, International Renewable Energy Agency, Footprint Data Foundation, United Nations Conference on Trade and Development, and OECD.

## 4 Empirical analysis

### 4.1 Computational results

**Fig. 1:**
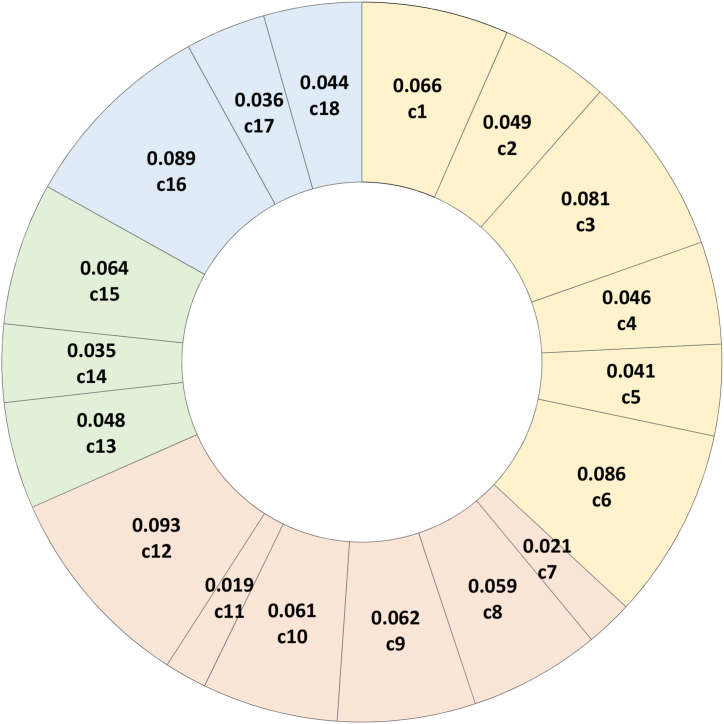
Weighting percentages of the indicators.

After obtaining the relevant data for the 18 indicators, the proposed evaluation framework is applied to dynamically evaluate the national green development levels of the G20 countries, following the eight steps presented in Section 3.1. The DEWM method through equations (1), (2), (3) is used to determine the weights of the indicators, as shown in Fig. 1. Among the indicators, the weights of GDP ($c_{12}$), number of patents granted ($c_{16}$), CO_2_ emissions ($c_{6}$), and share of renewable energy ($c_{3}$) are 9.30%, 8.90%, 8.60% and 8.10%, respectively, all exceeding 8% of the total weight.

The green development level rankings of each country in the G20 for 2015–2021 are calculated through equations (4), (5), (6), (7), (8), (9) and presented in Table 3. In Table 3, the top ranks indicate better green development level performance; countries with poorer green development levels are ranked lower. As shown in Table 3, some countries demonstrated slight changes in their rankings, whereas others remained stable. For instance, the US has consistently performed well, achieving first place from 2015 to 2021. Conversely, India consistently ranked at the bottom, except for being ranked second and last in 2018 and 2021, respectively. However, some countries exhibit significant changes throughout the study period. For example, the United Kingdom, which ranked third in 2016, fell to a lower position between 2017 and 2021. Overall, the rankings of most countries display both stability and variability, indicating that enhancing green development performance is a prolonged and challenging process.

**Table 3:** Ranking of green development level performance.

Country	2015	2016	2017	2018	2019	2020	2021
AR ($a_{1}$)	13	15	13	15	14	15	15
AU ($a_{2}$)	2	2	2	4	4	3	3
BR ($a_{3}$)	14	13	15	16	16	16	16
CA ($a_{4}$)	5	7	7	7	6	6	7
CH ($a_{5}$)	10	11	10	5	10	9	9
FR ($a_{6}$)	8	8	8	9	8	8	8
DE ($a_{7}$)	6	5	4	6	5	5	5
IN ($a_{8}$)	19	19	19	18	19	19	18
ID ($a_{9}$)	18	18	18	17	17	17	17
IT ($a_{10}$)	9	9	9	10	9	10	10
JP ($a_{11}$)	3	6	5	3	3	4	4
MX ($a_{12}$)	15	14	14	14	13	14	14
RU ($a_{13}$)	12	12	12	12	12	12	12
SA ($a_{14}$)	11	10	11	11	11	11	11
ZA ($a_{15}$)	17	17	17	19	18	18	19
KR ($a_{16}$)	4	4	3	2	2	2	2
TR ($a_{17}$)	16	16	16	13	15	13	13
UK ($a_{18}$)	7	3	6	8	7	7	6
US ($a_{19}$)	1	1	1	1	1	1	1

**Fig. 2:**
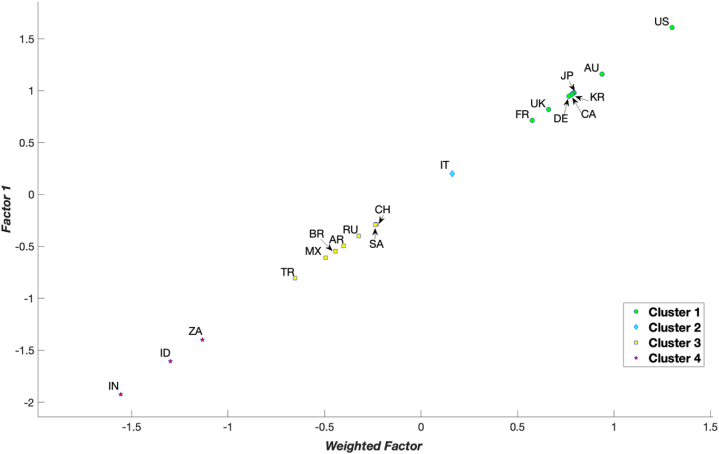
Clustering results for the G20 countries in 2015 by principle component analysis.

**Fig. 3:**
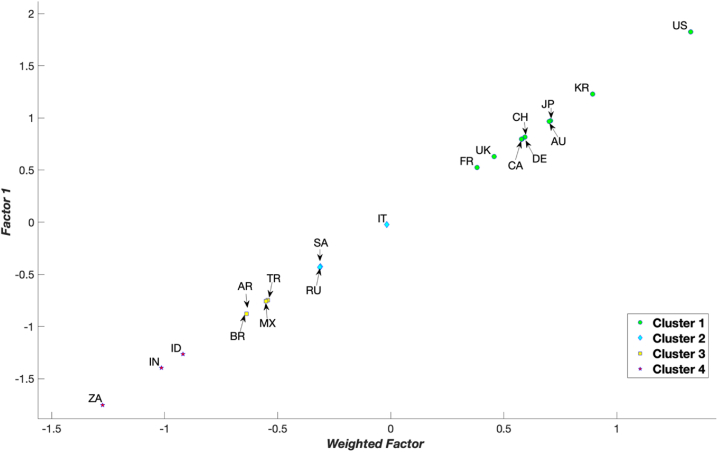
Clustering results for the G20 countries in 2018 by principle component analysis.

**Fig. 4:**
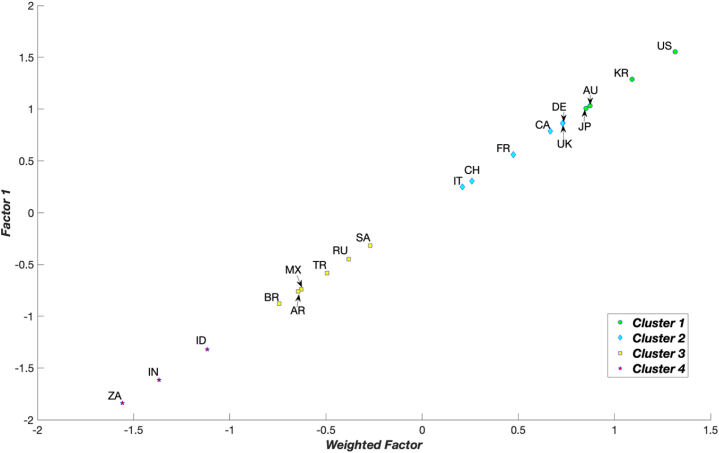
Clustering results for the G20 countries in 2021 by principle component analysis.

Considering that green development performance is a gradual process, each country needs to establish practical goals and reform directions based on appropriate benchmarks, namely, the standards of countries from which they can effectively learn. This can be accomplished through a clustering process that categorises countries into distinct clusters, enabling each country to identify a suitable reference – the best-in-class–within the corresponding cluster. Consequently, subsequent analyses utilised PCA as the clustering method (Section 3.1). In Fig. 2, Fig. 3, Fig. 4 and Table 4, the 19 G20 countries are grouped into four clusters. Cluster 1 represents countries with mature green development performance, whereas Cluster 4 comprises countries in the early stages of green development performance. From Fig. 2, Fig. 3, Fig. 4 and Tables 4 and it can be concluded that four countries––the US, Australia, the Republic of Korea, and Japan––are consistently positioned in Cluster 1 for all years from 2015 to 2021. This indicates that these countries are leading in green development performance and have reached a mature level. Conversely, three countries––India, Indonesia, and South Africa––were primarily in the early stages of green development performance during 2015–2021.

**Table 4:** Clustering of the G20 countries.

2015		2016		2017		2018		2019		2020		2021	
Country	Cluster	Country	Cluster	Country	Cluster	Country	Cluster	Country	Cluster	Country	Cluster	Country	Cluster
US	1	US	1	US	1	US	1	US	1	US	1	US	1
AU	1	AU	1	AU	1	KR	1	KR	1	KR	1	KR	1
JP	1	UK	1	KR	1	JP	1	JP	1	AU	1	AU	1
KR	1	KR	1	DE	1	AU	1	AU	1	JP	1	JP	1
CA	1	DE	1	JP	1	CH	1	DE	1	DE	1	DE	2
DE	1	JP	1	UK	1	DE	1	CA	1	CA	1	UK	2
UK	1	CA	1	CA	1	CA	1	UK	1	UK	1	CA	2
FR	1	FR	1	FR	1	UK	1	FR	1	FR	1	FR	2
IT	2	IT	2	IT	2	FR	1	IT	2	CH	2	CH	2
CH	3	SA	2	CH	2	IT	2	CH	2	IT	2	IT	2
SA	3	CH	2	SA	2	SA	2	SA	2	SA	2	SA	3
RU	3	RU	2	RU	3	RU	2	RU	3	RU	2	RU	3
AR	3	BR	3	AR	3	TR	3	MX	3	TR	3	TR	3
BR	3	MX	3	MX	3	MX	3	AR	3	MX	3	MX	3
MX	3	AR	3	BR	3	AR	3	TR	3	AR	3	AR	3
TR	3	TR	3	TR	3	BR	3	BR	3	BR	3	BR	3
ZA	4	ZA	4	ZA	4	ID	4	ID	4	ID	4	ID	4
ID	4	ID	4	ID	4	IN	4	ZA	4	ZA	4	IN	4
IN	4	IN	4	IN	4	ZA	4	IN	4	IN	4	ZA	4

### 4.2 Robustness Check of the results

To confirm the robustness and feasibility of the proposed dynamic evaluation DEWM–DTOPSIS–PCA framework, a sensitivity analysis and a comparative analysis are conducted. It is essential to assess the change in weight values because the weights of the indicators significantly affect the rankings [37,38]. Accordingly, a sensitivity analysis is performed to account for variations in indicator weights. Three scenarios are generated and analysed for this purpose. Scenario 1, the baseline scenario, uses the indicator weights derived from the DEWM method, as presented in Subsection 4.1. Scenario 2 involves assigning equal weights to all indicators. Scenario 3 assigns equal weights to benefit indicators that need to be maximised while setting the weight to zero for ineffective indicators that should be minimised [37]. In this study, the benefit indicators include Forest cover ($c_{1}$), Arable land ($c_{2}$), Share of renewable energy ($c_{3}$), Ecological footprint ($c_{4}$), Gross national income ($c_{7}$), Agriculture, forestry and fishing, value added ($c_{8}$), Foreign direct investment ($c_{9}$), Gross fixed capital formation ($c_{10}$), GDP per capita ($c_{11}$), GDP ($c_{12}$), Life expectancy ($c_{13}$), Human development index ($c_{14}$), Number of patents granted ($c_{16}$), Research and development expenditure ($c_{17}$), Innovation ability/high technology exports ($c_{18}$), whereas the cost indicators are the Energy intensity level of primary energy ($c_{5}$), CO_2_ emissions ($c_{6}$), and Unemployment rate ($c_{15}$). The sensitivity analysis results for the proposed DEWM–DTOPSIS–PCA framework, using these criteria weights in the respective scenarios, are presented in Table 5 for the years 2015, 2018, and 2021, owing to space limitations. The results indicate that the US consistently performed well, securing first place in the ranking across all scenarios. Other countries show variations, with moderate changes in their overall ranking positions across different scenarios. Thus, the overall sensitivity results demonstrate that the proposed framework is relatively robust when considering the indicator weights that are sensitive to the final ranking.

**Table 5:** Results of the sensitivity analysis with three scenarios.

Country	2015			2018			2021		
	Scenario 1	Scenario 2	Scenario 3	Scenario 1	Scenario 2	Scenario 3	Scenario 1	Scenario 2	Scenario 3
AR	13	18	15	15	17	19	15	11	16
AU	2	2	3	4	9	9	3	4	5
BR	14	10	14	16	18	18	16	17	14
CA	5	3	2	7	7	6	7	6	9
CH	10	9	10	5	3	4	9	10	6
FR	8	11	4	9	6	7	8	8	8
DE	6	8	5	6	5	3	5	5	3
IN	19	19	18	18	19	14	18	14	18
ID	18	14	16	17	15	16	17	18	15
IT	9	7	6	10	13	11	10	12	11
JP	3	6	7	3	4	2	4	2	4
MX	15	15	13	14	12	15	14	16	17
RU	12	16	11	12	10	12	12	13	10
SA	11	12	12	11	14	13	11	15	12
ZA	17	17	19	19	16	17	19	19	19
KR	4	5	8	2	2	5	2	3	2
TR	16	13	17	13	11	8	13	9	13
UK	7	4	9	8	8	10	6	7	7
US	1	1	1	1	1	1	1	1	1

Investigating the results of the MCDM approach is crucial to validate and ensure their reliability [38]. Conducting a comparative analysis is a standard practice among researchers to address validation and reliability issues, thereby mitigating the risk of potentially misleading results [39]. Thus, to verify the grouping procedure, hierarchical cluster analysis (HCA) [9,40] and k-means [41], which are widely used clustering methods, are employed for comparison, as they have been proven to be effective grouping methods. Table 6 presents the clustering comparison results of the three clustering methods. Cluster 1 represents countries with mature green development performance, whereas Cluster 4 comprises countries in the early stages of green development performance. For 2018 and 2021, the three methods produce similar clustering assignments for the best clusters. It can be concluded that the four countries––the US, Australia, the Republic of Korea, and Japan––are consistently positioned in Cluster 1 using the three clustering methods. Although there are some differences in the assignments to Clusters 2 and 3, countries with similar levels of green development are still clustered together. Moreover, the clustering results obtained using the PCA method are highly consistent with those derived using the HCA and k-means for 2015, 2018, and 2021 for Cluster 4 which includes three countries––India, Indonesia, and South Africa––that are primarily in the early stages of green development performance. Therefore, PCA exhibits the same accuracy and validity as the HCA and k-means methods, ensuring the robustness of the proposed method and verifying that countries can feasibly apply the proposed dynamic framework to determine appropriate benchmarks for green development performance levels.

**Table 6:** Comparison of Clustering across different Clustering methods.

2015				2018				2021			
Country	PCA	HCA	k means	Country	PCA	HCA	k means	US	1	HCA	k means
US	1	1	1	US	1	1	1	US	1	1	1
AU	1	1	1	KR	1	1	1	KR	1	1	1
JP	1	1	1	JP	1	1	1	AU	1	1	1
KR	1	1	1	AU	1	1	1	JP	1	2	2
CA	1	1	1	CH	1	1	1	DE	2	2	2
DE	1	1	1	DE	1	1	1	UK	2	2	2
UK	1	2	2	CA	1	1	1	CA	2	2	2
FR	1	2	2	UK	1	1	1	FR	2	2	2
IT	2	2	2	FR	1	2	2	CH	2	2	2
CH	3	3	3	IT	2	2	2	IT	2	2	2
SA	3	3	3	SA	2	2	2	SA	3	2	3
RU	3	3	3	RU	2	3	2	RU	3	3	3
AR	3	3	3	TR	3	3	3	TR	3	3	3
BR	3	3	3	MX	3	3	3	MX	3	3	3
MX	3	3	3	AR	3	3	3	AR	3	3	3
TR	3	3	4	BR	3	3	3	BR	3	3	3
ZA	4	4	4	ID	4	4	4	ID	4	4	4
ID	4	4	4	IN	4	4	4	IN	4	4	4
IN	4	4	4	ZA	4	4	4	ZA	4	4	4

## 5 Conclusion and policy implications

As a group of major and dynamic global economies, the G20 is actively pursuing sustainable development to achieve the United Nations Sustainable Development Goals by 2030. Despite these efforts, the G20 members continue to face significant environmental protection challenges. In pursuit of sustainable economic growth, G20 member states have shown substantial interest in advancing green development. However, some G20 member states seem to be underperforming in their progress towards green development.

This study conducted a dynamic evaluation of the green development performance of G20 countries for the period 2015–2021, constrained by data availability. An assessment indicator system was developed encompassing environmental, economic, social, and innovation dimensions to meet the requirements for evaluating green development performance. To conduct this dynamic evaluation, this study proposed a framework incorporating the DEWM, DTOPSIS, and PCA methodologies. This evaluation framework involved assigning weights to the indicators influencing national green development performance, followed by ranking and clustering the countries accordingly.

It is evident from the results for the evaluation indicators that GDP, number of patents granted, CO₂ emissions, and share of renewable energy are the most significant indicators within the evaluation framework. These findings suggest that these indicators are the primary determinants of national green development performance levels.

According to the ranking evaluation and clustering analysis, the United States, Australia, Republic of Korea, and Japan have consistently demonstrated strong green development performance among G20 members from 2015 to 2021, securing top positions in the rankings. This result was expected, as these countries have strong economic foundations and prioritise the coordinated development of their economies, societies, environments, and innovations. Moreover, countries with similar characteristics, such as economic growth and innovation implementation, tend to cluster within specific ranges on the ranking list. For example, the United States, Australia, Republic of Korea, and Japan are less reliant on manufacturing and more dependent on scientific knowledge and skills. Over the past several decades, these countries have introduced new and improved versions of environmental technology through collaborative efforts and strategic partnerships [42]. Conversely, India, Indonesia, and South Africa are in the early stages of green development during various periods of the same timeframe. This trend was expected because these nations face significant challenges, particularly concerning economic and environmental issues. These findings align with the previous research by Stevens [43], who demonstrated that these countries struggle with fossil fuel dependency and emission-intensive industries, negatively impacting the stringency of their mitigation policies.

The results of this study suggest several actionable proposals to enhance the sustainability of green development among the G20 nations. First, governments should focus on economic growth as a key driver of future green development performance by aligning GDP growth with sustainable practices to create a robust foundation for environmental progress. Policymakers should develop strategies that foster innovation and increase patent applications by prioritising investments in R&D programs to drive technological advancements that support green development. Additionally, refining and developing policies and technologies to advance low-carbon standards is essential, and G20 leaders should coordinate with appropriate institutions to ensure sustainable growth while protecting natural resources and environmental assets, such as temperate climates. Governments should also emphasise renewable energy strategies within a circular green economy framework to achieve sustainable development, encouraging G20 member nations to collaborate in transferring and utilising renewable energy technologies. Moreover, raising community awareness and increasing societal demand for green energy solutions while reducing reliance on fossil fuels is crucial for facilitating energy transition. To this end, the G20 governments should adopt sustainable approaches to economic development and establish clear pathways to achieve these objectives by integrating sustainability into economic policies and initiatives. Finally, the G20 should create knowledge-sharing platforms to disseminate best practices and guidelines from countries with advanced green development, such as the United States, to support nations with lower levels of green development and overcome environmental challenges while pursuing economic growth.

This study makes significant contributions to both the research community and the practical green development efforts of G20 members in several key areas. First, it introduces a novel dynamic evaluation framework that integrates the DEWM, DTOPSIS, and PCA to assess green development in a dynamic context. Second, the proposed framework serves as a benchmarking tool for evaluating the green development of the G20 countries, providing a foundation for future policy formulation and implementation. Finally, the framework presented in this study is versatile, broadly applicable, and not significantly constrained by geographical or thematic considerations.

This study has some limitations that suggest potential avenues for future research. First, the proposed evaluation framework was developed within specific contexts and did not address the inherent uncertainties and ambiguities in the evaluation process. Future research should explore the application of fuzzy set theory to investigate this topic in uncertain environments. Second, although the proposed evaluation framework provides a comprehensive overview of the benchmark results based on weighted indicators, it does not consider the interrelationships among these indicators. Future studies should elucidate the interconnections between various indicators and their implications for evaluating developmental performance. Third, the construction of an assessment indicator system was constrained by data availability. Despite certain indicators effectively capturing a country's green development level, some may be excluded owing to insufficient data collection, such as managed waste or social security metrics. These limitations underscore the critical importance of data as the most objective standard and fundamental basis for research, highlighting the need for governments to prioritise data collection efforts. Finally, further comparative and sensitivity analyses are required.

## Funding

This work was funded by the Deanship of Scientific Research, Vice Presidency for Graduate Studies and Scientific Research, 10.13039/100019224King Faisal University, Saudi Arabia (the Annual Grants 2024) under Grant No. KFU24.

## Data availability statement

Data will be made available on request.

## CRediT authorship contribution statement

**Tarifa Almulhim:** Writing – review & editing, Writing – original draft, Supervision, Methodology, Investigation, Funding acquisition, Formal analysis, Data curation, Conceptualization.

## Declaration of competing interest

The authors declare that they have no known competing financial interests or personal relationships that could have appeared to influence the work reported in this paper.

## Footnotes

1 G20 member countries, except for those in the European Union: Argentina (AR), Australia (AU), Brazil (BR), Canada (CA), China (CH), France (FR), Germany (DE), India (IN), Indonesia (ID), Italy (IT), Japan (JP), Republic of Korea (KR), Mexico (MX), Russia (RU), Saudi Arabia (SA), South Africa (ZA), Turkey (TR), United Kingdom (UK) and United States (US).
